# Face expectancy cues differentially modulate conflict processing driven by emotional incongruence: an EEG study

**DOI:** 10.1038/s41598-025-34447-9

**Published:** 2026-01-10

**Authors:** Maria Pires Coelho, Daniel Agostinho, Teresa Sousa, Gabriel Pires, Miguel Castelo-Branco

**Affiliations:** 1 Coimbra Institute for Biomedical Imaging and Translational Research (CIBIT), University of Coimbra, Coimbra, Portugal; 2 Institute for Nuclear Sciences Applied to Health (ICNAS), University of Coimbra, Coimbra, Portugal; 3 Institute of Physiology, Faculty of Medicine, University of Coimbra (FMUC), Coimbra, Portugal; 4 Institute of Systems and Robotics, University of Coimbra, Coimbra, Portugal; 5 Centre for Informatics and Systems, University of Coimbra (CISUC), Coimbra, Portugal; 6 Engineering Department, Polytechnic Institute of Tomar, Tomar, Portugal; 7 Intelligent Systems Associate Laboratory (LASI), Guimarães, Portugal; 8 Pólo das Ciências da Saúde, University of Coimbra, Azinhaga de Santa Comba, Coimbra, 3000-548 Portugal

**Keywords:** Conflict processing, Emotional processing, Event-related potential, Expectancy, Neuroscience, Psychology, Psychology

## Abstract

Adaptive behavior in social interactions requires the effective processing of conflicting emotional information. The impact of expectancy on conflict processing remains a relevant research question. Here we investigated the influence of primed expectancy on conflict processing. To achieve this goal, we used the Emotional Stroop paradigm and variants where expectancy was introduced using facial expression or emotional letter labels. Neurophysiological and behavioral data were collected from 20 healthy participants who completed these three conditions (in the presence or absence of prior expectancy cues). We examined conflict-related components N450 and the Conflict Slow Potential in the classical Emotional Stroop condition and in conditions with manipulated expectancy. When expectancy was introduced, we found a significant effect on conflict processing, with a difference between face and letter emotion cues. Parietal alpha and beta power decreases occurred specifically for face expectancy cues and changed only minimally during conflict, whereas label-based expectancy was associated with less desynchronization during expectancy and a stronger alpha/beta decrease during conflict. These findings suggest that attentional resources are differently prioritized by face versus letter emotion expectancy cues, with face-driven expectancy generating distinctive neurophysiological patterns and a potential facilitation of subsequent conflict resolution.

**Supplementary Information:**

The online version contains supplementary material available at 10.1038/s41598-025-34447-9.

## Introduction

### Expectancy effect of emotional conflict processing

Conflict processing refers to cognitive mechanisms involved in detecting and resolving inconsistent information^1^. Given its role in guiding adaptive learning and goal-directed behavior^2^, incongruence processing is a critical facet of everyday conflict handling^3,4^. While previous research has addressed the impact of affective and emotional processing on incongruence processing^3,5,6^, the role of expectation, which is typically present in socioemotional interactions^7^ remains to be understood. Investigating how expectancy cueing can modulate incongruence processing offers a new window into improved understanding of conflict processing itself. Here, we thus seek to investigate the neural mechanisms underlying the interplay between these processes.

### Neural correlates of emotional conflict processing

Previous event-related potential (ERP) studies have addressed the distinct neurophysiological underpinnings of conflict processing in emotional contexts, such as the centroparietal N450^8^ (also often referred to as the N400^9^ in broader contexts), where more negative amplitudes are evident in incongruent stimuli than in congruent stimuli, and the conflict slow potential (CSP)^8^ (also referred to as the late positive component^10^, or sustained potential^11^ shown from 500 ms onwards after stimulus onset, showing a divergence in amplitude between congruent and incongruent stimuli in centroparietal channels and linked to conscious processing. Whilst ERPs are often studied in this context, oscillatory markers have not been as much of a focus of study. Previous research has associated increased parietal alpha power with the suppression of task-irrelevant information, therefore optimizing attentional focus and aiding in conflict resolution^12^. Parietal beta power modulations have frequently been linked to top-down attentional control processes, with decreases in beta power reflecting the allocation of attentional resources during visual processing^13,14^. Previous findings have also reported parietal beta power decreases during post-cue periods^15^. Van Wijk et al. (2009) found that beta power decreased significantly less when a cue contained no useful information about the subsequent action^16^, suggesting that these modulations may also contribute to stimulus anticipation and expectancy creation.

Expectancy can be introduced in an experimental design using stimulus onset asynchrony paradigms^17^. By manipulating stimulus anticipation over an extended period, it is possible to create an expectancy effect which modulates perceptual and attentive stimulus processing. Different types of information may differentially shape these expectancy-related processes, as perceptually salient stimuli such as faces engage specialized visual mechanisms and attract top-down attention more strongly than symbolic or linguistic cues^14,18^. However, the extent to which such face-driven expectations influence subsequent conflict processing remains unclear.

### Study overview and hypotheses

We hypothesized that primed expectations, particularly face-driven expectations, have a differential impact on conflict resolution, and this was investigated by a variation of the Emotional Stroop task at both behavioral and neurophysiological levels.

Accordingly, to investigate how expectations affect conflict processing in emotion recognition we assessed the effects of two distinct primed expectations: face-driven and letter label-driven on conflict resolution, using an Emotional Stroop design. Emotional Stroop Paradigms offer a unique approach for studying how emotional interference may influence conflict resolution^3^. By employing facial expressions to elicit emotional recognition responses, relevant insights were provided on how conflict is processed under emotional contexts^19^.

We analyzed ERP components linked to conflict processing and resolution, namely the N450 and CSP, and examined how these were modulated by expectancy as a function of cue type. In addition, we investigated how expectancy influenced oscillatory activity, focusing on parietal theta, alpha and beta modulations.

We hypothesized that parietal beta activity would reflect changes in top-down control processes, supporting the maintenance of task-relevant expectations that facilitate conflict resolution, whereas alpha activity would index sensory and inhibitory mechanisms, modulating shifts in visual processing to suppress irrelevant input and optimize perceptual processing of anticipated stimuli. Theta power was additionally examined in an exploratory manner, given its proposed role in cognitive control and conflict monitoring. By examining these neurophysiological patterns alongside behavioral performance, we aim to provide a deeper understanding of how expectancy can modulate conflict processing in socioemotional contexts involving face processing.

## Methods

### Participants

Twenty healthy participants (9 females, 11 males) with a mean age of 24 years (standard deviation (SD) = 2.92) were recruited using snowball sampling. Participants were Portuguese adults, 18 years old or older, with normal or corrected vision. The sample size was determined based on previous EEG studies employing similar paradigms and within-subject designs^18,20^. A sensitivity analysis in G*Power 3.1^21^ (F tests, repeated-measures ANOVA, within factors; *α* = 0.05, power = 0.90, *N* = 20) for our 3 (Expectancy) × 2 (Congruency) design indicated a minimum detectable effect size of f = 0.309 (partial *η²* ± 0.087). Exclusion criteria included individuals with a history of neurological, psychiatric, or neurodevelopmental disorders as well as those scoring below the cut-off normal standard deviation (within 1 SD of the mean) on the matrix reasoning (M = 23.3 ± 1.9) and vocabulary (M = 48.5 ± 7.1) subtests of the Portuguese version of the Wechsler Adult Intelligence Scale-III (WAIS-III)^22^. All participants provided written informed consent, and the study was approved by the Ethics Committee of the Faculty of Medicine of the University of Coimbra (*Comissão de Ética da Faculdade de Medicina da Universidade de Coimbra*; approval ID: CE-001/2021). This study followed the ethical guidelines of the Declaration of Helsinki (2013 revision) to protect the rights and well-being of all participants.

### Experimental design

**Fig. 1:**
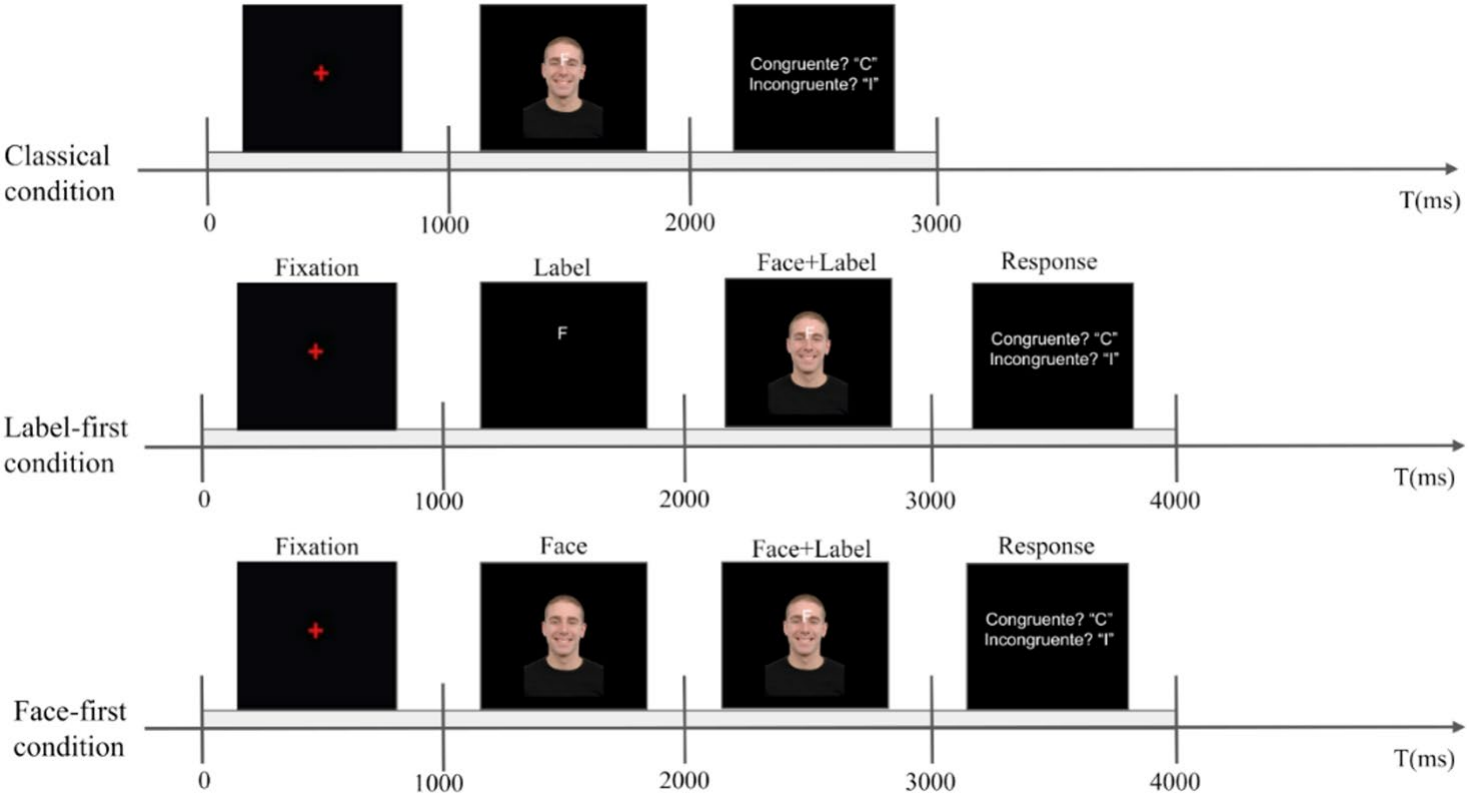
Experimental task. A variation of the face-word emotional Stroop task is presented. In the Classical condition (CL), the face and the label appear simultaneously. In the Label-first condition (LF), the label appears first, followed by the face, with the label overlapping the face. Finally, in the Face-first condition (FF) the face appeared first, followed by the face and the label overlapping. In both the LF and the FF conditions, expectancy is generated through stimulus anticipation. All faces were retrieved from the open access radboud faces database^25^.

**Fig. 2:**
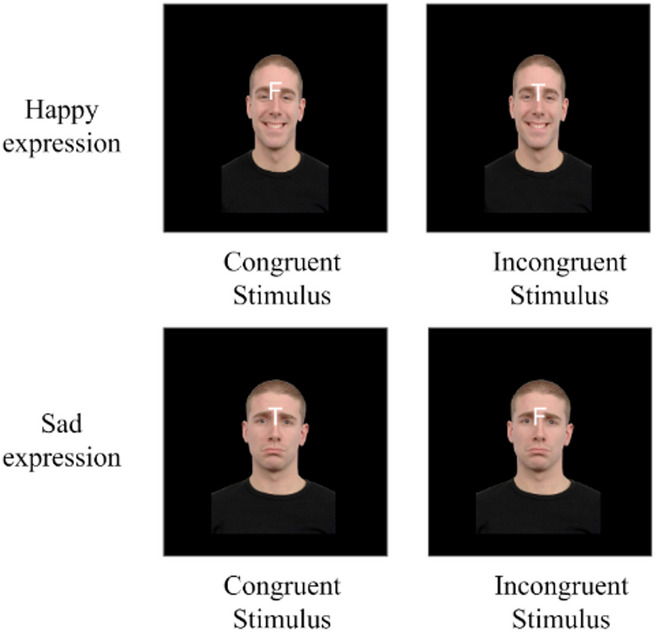
Congruent and incongruent examples. On the left side, congruent stimuli are presented, with a happy face and a happy label (top) and a sad face with a sad label (bottom). On the right panel, incongruent stimuli are shown. A happy face with a sad label is presented in the top corner, and a sad face with a happy label is presented in the bottom corner.

The experimental task employed a face-word Emotional Stroop paradigm implemented in MATLAB^23^ using the Psychophysics Toolbox Version 3 (PTB-3)^24^. Our task involved the presentation of 6 facial expressions (3 male, 3 female) retrieved from the Radboud Faces Database^25^ representing happiness or sadness (with visual angles of approximately 9.46° in width and 14.74° in height), with superimposed text labels (either “T” for “triste” or “F” for “feliz”, meaning sad and happy, respectively, with visual angles of approximately 0.95° in width and 1.91° in height), positioned centrally between the eyes, that were congruent or incongruent with the expressed facial expression (Figs. 1 and 2) in a 24-inch screen (1920 × 1080 pixels, refresh rate = 60 Hz). The proportions of congruent and incongruent stimuli were balanced. Three conditions were tested to investigate the influence of the stimulus-primed expectancy. In classical condition (CL), face and emotion labels appeared simultaneously. For the label-first condition (LF), the letter label appeared first, followed by the face, with the label overlapping the face. In the face-first condition (FF), the face appeared first, followed by face and label overlapping.

Participants were instructed to identify whether a trial was congruent or incongruent by pressing the corresponding key (letter C or I on the keyboard). The experiment was presented on a black background, with stimuli centrally displayed to minimize visual variability and facilitate stable fixation. A red fixation cross (40 pixels; ± 1.91° visual angle) appeared at the center of the screen. Face stimuli were shown within a fixed 275 × 400 pixel rectangle, centered horizontally and positioned slightly below the fixation cross (± 50 pixels). The response labels (“C” and “I”) were displayed in white (Arial, size 48), symmetrically positioned around the center of the screen. Participants were instructed to maintain central fixation throughout the task. Although eye movements were not recorded, the central presentation and fixation instruction likely reduced systematic oculomotor contributions. A short practice session was conducted before the main experiment to ensure that the participants understood the task completely. In the CL condition, each trial lasted 3 s, while in the LF and FF conditions, it was extended to 4 s to allow for the expectancy period. Regardless of the condition, every trial commenced with the red fixation cross lasting 1 s, to enhance participants’ attentional focus. The fixation cross was followed by the presentation of the congruent or incongruent conditions lasting for 1 s, which was preceded in LF and FF trials by expectancy creation (1-second long). Following the stimulus onset, a black screen appeared with a response prompt indicating the key press option: “Congruent? “C”; Incongruent? “I”. Participants were instructed to respond only after the question appeared to prevent motor artifacts from affecting the neurophysiological conflict-related signals.

The experimental implementation was divided into eight runs, each lasting approximately 6 min, and consisted of 96 trials equally distributed across conditions. The order of the trials was fully randomized within each run to prevent predictability. The total duration was approximately 2 h, including the previously mentioned WAIS-III subtests as a cognitive assessment (20 min), EEG preparation (30 min), data acquisition (60 min). All participants performed the task in a standardized, quiet, and distraction-free environment to minimize potential confounding factors that could influence their results. The full task implementation can be found at https://github.com/Daniel-Agostinho/ESP.

### Data acquisition

Data were collected at the Institute for Nuclear Sciences Applied to Health (ICNAS) in Coimbra. Participants were administered the WAIS-III^22^ vocabulary and matrix subtests as inclusion criteria. Electroencephalographic (EEG) activity was recorded using an actiCHamp amplifier (Brain Products GmbH, Gilching, Germany). A 64-channel EEG cap was used, with Ag/AgCl active electrodes placed on the scalp (ActiCHamp Plus, Brain Products GmbH, Gilching, Germany). EEG electrodes were positioned according to the 10–20 international system, standardizing the electrode placement for scalp recording. Data were collected at a sampling rate of 1000 Hz. The reference electrode was placed at the left mastoid, while the vertical and horizontal electrooculogram (EOG) electrodes were placed above and below the left eye, as well as to the left of the left eye and to the right of the right eye. The EOG signal was recorded to identify visual artifacts and facilitate their removal. Impedances were kept below 10 kΩ to ensure adequate signal quality. Data were recorded using BrainVision recorder software^26^.

### Behavioral data processing analyzes

Trials with omitted responses were treated as errors and excluded from the analyses. A detailed description of omissions count per participant per condition is available in the Supplementary Material, Table S1. Following exclusion of invalid responses, the mean number of valid trials retained per participant was ± 254 for CL (99.01% of total trials), ± 253 for LF (98.36% of total trials) and ± 254 for FF (98.85% of total trials). Reaction times (RT) were not analyzed, as responses were intentionally withheld until the response prompt. Descriptive RT statistics (means and standard deviations by condition) are reported in Supplementary Analysis S2. Accuracy was calculated per participant based on the percentage of congruent and incongruent correct responses. The percentages of correct responses for each of the three tested conditions (classical, face-first, and label-first conditions) were extracted and compared across conditions.

### Neurophysiological processing and analyzes

EEG signal preprocessing and analyses were performed using the EEGLAB (version 2023.0) toolbox^27^ in MATLAB^23^. For offline preprocessing of EEG data, a pipeline was created with the necessary steps according to Makoto’s preprocessing guidelines^28^.

First, the data were downsampled to a frequency of 500 Hz. A high-pass filter was then applied at 0.1 Hz, and a low-pass filter was applied at 40 Hz. After these steps, noisy channels (channels with electrical interference or a very low signal-to-noise ratio) were identified by visual inspection and interpolated based on data from neighboring channels, and the data were re-referenced to their average to provide a clearer representation of the brain’s electrical activity. Following this step, independent component analysis (ICA) was performed to remove eye and motor artifacts, electrode drift, or any abnormal pattern identified^27^. Finally, the data were segmented into epochs of interest, from − 2000 ms to 1000 ms for six conditions (the three main conditions divided by congruent and incongruent events), locked to the moment where the face and the label were overlapping for every condition.

#### Event-related potentials – N450 and CSP

All epochs were normalized by subtracting the common baseline moment, referring to the last 500 ms of the fixation cross (−500 ms to 0 ms for CL, and − 1500 ms to −1000 ms for LF and FF). At least 90 trials resulted per condition. ERP analyses focused primarily on a central channel cluster (C1, C2, Cz), and on a parietal channel cluster (P1, P2, Pz) as a complementary, subsequent analyzes.

For the N450 and the CSP, the mean amplitude between 350 and 450 ms and 600 ms to 800 ms, respectively, was extracted in the central cluster^29,30^. The latency windows were defined a priori based on prior ERP literature^30–33^. Mass-univariate analyses controlled by cluster-based permutation tests were conducted over the full epoch to confirm condition-related effects across time and scalp regions, and CSP was then extracted in parietal sites^29,34^ from 600 ms to 800 ms.

#### Event-related spectral perturbation

Event-related Spectral Perturbation (ERSP) analysis was conducted in EEGLAB^27^ to investigate neural oscillatory dynamics during the formation of primed expectancy and subsequent conflict processing. Frequency power variations over time were calculated using wavelet decomposition as the default spectral decomposition method. EEG data from the parietal cluster were analyzed for two periods: primed expectancy creation (−1000 ms to 0 ms) and for conflict resolution (0 ms to 700 ms), within a frequency range from 4 Hz to 40 Hz.

Based on prior work linking alpha and beta oscillations to attentional and monitoring processes^13,14^, frequency analyses focused a priori on alpha and beta bands, with theta band power examined in a complementary, exploratory analysis. For each trial and participant, ERSPs were computed with respect to a baseline defined as the 500 ms following the onset of the fixation cross, and mean power was extracted for theta, alpha, and beta bands in each of the two time windows, [−600 ms, −400 ms] and [400 ms, 600 ms] corresponding to expectancy and conflict, respectively. Since previous literature had not established consistent time–frequency markers of stimulus anticipation^14,20^, the temporal windows corresponding to expectancy and conflict processing were defined based on data-driven inspection of the grand-average spectral patterns across conditions.

#### Source localization analyzes

For source localization analysis, we used standardized low-resolution brain electromagnetic tomography (sLORETA)^35,36^. The intracerebral volume was segmented into 6239 voxels (5-mm resolution) and solved on the MNI152-T1 template. The standardized current density for each voxel was estimated. sLORETA standardizes the solution, so there is no user-set regularization and voxel values are comparable. Supporting evidence from EEG/fMRI studies confirm the accuracy of source estimation using sLORETA^37^, as well as studies providing reliable results without localization bias^38^.

### Statistical analysis

Descriptive analyses were performed to characterize the participants by sex, age, laterality index, and education. The effects of experimental cueing conditions (CL, LF, and FF) and conflict (congruent vs. incongruent) and the possible interaction between them on the accuracy were analyzed through a two-way Repeated Measures ANOVA. Greenhouse-Geisser correction was applied to adjust the degrees of freedom in the repeated-measures ANOVA. Effect sizes were measured using partial eta squared (*partial η²*). A trend analysis was also performed through a linear regression to explore any systematic patterns across the levels of the independent variables.

Electrophysiological data (ERPs and ERSPs) were analysed using linear mixed-effects models (LMMs), complemented by a mass-univariate analysis and source localisation. For ERPs, hypothesis-driven analyses first focused on the central cluster, where separate LMMs were fitted for the N450 and CSP windows. Fixed effects included Condition (CL, LF, FF), Congruency (congruent, incongruent), and their interaction, with Run (1–8) and Emotion (happy, sad) as covariates, and a random intercept for participants to model between-subject variability.

To identify where in time and scalp space expectancy-related effects were strongest, a mass-univariate analysis controlled for by cluster based permutation was conducted in LIMO EEG. A two-way repeated-measures ANOVA with Condition (LF, FF) and Congruency was estimated at each electrode and time point and tested using a cluster-based permutation framework with TFCE (Threshold Free Cluster Enhancement), providing familywise error control at *α* = 0.05 over the full epoch and scalp electrode distribution.

Parietal ERPs were then examined using LMMs applied to CSP windows at posterior sites, with the same fixed-effect structure (Condition, Congruency, Condition × Congruency, Run, Emotion) and random intercepts for participants. For ERSPs, LMMs were specified on parietal mean power values with Condition (LF, FF), Period (expectancy, conflict), and Band (theta, alpha, beta) as fixed factors, and Congruency, Run, and Emotion as additional covariates. Follow-up models were then ran separately for each band, focusing on the Condition × Period interaction. For all LMMs, estimated marginal means and Bonferroni-corrected pairwise comparisons were used to characterise significant main effects and interactions.

For key non-significant contrasts, Bayesian paired-samples t-tests were computed on the mean amplitudes to distinguish between evidence for the absence of an effect and a lack of sensitivity. Exploratory Pearson correlation coefficients were computed between accuracy in the label-first and face-first conditions and parietal oscillatory indices (alpha/beta power change from expectancy to conflict). For these correlations, Bayesian Pearson tests were also run to obtain Bayes factors BF₀₁ (null vs. alternative), to quantify the degree of evidence for or against the presence of an association.

For source-localised activity, sLORETA’s statistical non-parametric mapping with 5000 permutations on paired data was used. The voxelwise statistic was the signed log F-ratio (lnF), where the sign encodes direction (negative = LF > FF; positive = FF > LF). Multiple comparisons were controlled at *p* = 0.05 family-wise error (FWER) using permutation-derived critical values, and reported results include the FWER-corrected peak voxel (MNI coordinates and lnF value).

## Results

### Behavioral data

Descriptive statistics for behavioral performance in each condition are presented in Table S3 in the supplementary material. No significant differences were found in accuracy between conditions (F(2) = 2.844, *p* = 0.0741, *partial η²* = 0.105), nor did the presence of conflict significantly influence accuracy (F(1) = 0.458, *p* = 0.507, *partial η²* = 0.024). Moreover, no interaction was found between the type of condition and congruence (F(2) = 0.906, *p* = 0.413, *partial η²* = 0.046).

**Fig. 3:**
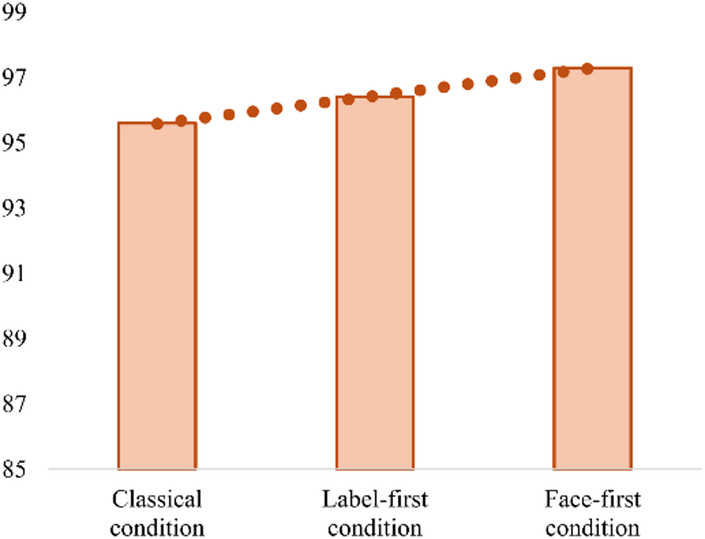
Trend analysis of behavioral response accuracy (%). A significant condition-related main effect was found. Specifically, the Face-first condition showed significantly higher accuracy (M = 97.30, SD = 3.25) compared to both the label-first condition (M = 96.40, SD = 3.71) and the Classical Emotional Stroop condition (M = 95.60, SD = 4.00).

Nevertheless, further trend analysis revealed a significant main condition-related effect. Specifically, the FF condition showed significantly higher accuracy rates (M = 97.30, SD = 3.25) than both the LF (M = 96.40, SD = 3.71) and CL conditions (M = 95.60, SD = 4.00) (F(1) = 5.885, *p* = 0.017), with a moderate effect size (R² = 0.048) (Fig. 3).

### Event-related potentials related to conflict processing

The first question addressed whether conflict-related evoked responses were present in the classical Emotional Stroop (CL) conditions and in the conditions in which expectancy was introduced (LF and FF).

#### N450 and CSP modulation by conflict resolution

**Fig. 4:**
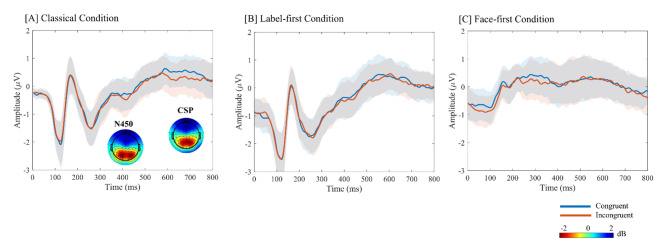
Central N450 and CSP in the three cueing conditions. Grand-average ERPs at the central cluster (C1, C2, Cz) for the Classical (CL), Label-first (LF), and Face-first (FF) conditions. Time 0 ms marks the conflict onset, corresponding to the face–label pair. Blue and orange traces show congruent and incongruent trials, respectively; the shaded regions represent the standard error (SE) of the mean across participants. In panel [**A**] (Classical condition), scalp topographies for the N450 (350 ms to 450 ms) and CSP (600 ms to 800 ms) windows are shown. Mean amplitudes in these predefined windows were used in the linear mixed-effects models reported above.

Central N450 and CSP amplitudes were analyzed using linear mixed-effects models. A first model was restricted to the classical condition (CL) and included Congruency (congruent, incongruent), Run, and Emotion as fixed effects. For the N450 window (350–450 ms), this model revealed no main effect of Congruency (F(1, 636) = 0.96, *p* = 0.33), and no significant effects of Run (F(1, 636) = 2.85, *p* = 0.09) or Emotion (F(1, 636) = 0.01, *p* = 0.94). Similarly, for the CSP window (600–800 ms), Congruency did not reach significance (F(1, 636) = 1.36, *p* = 0.24), nor did Run (F(1, 636) = 0.04, *p* = 0.84) or Emotion (F(1, 636) = 0.37, *p* = 0.54) (Fig. 4A). Bayesian paired-samples t-tests comparing incongruent vs. congruent mean amplitudes in the N450 and CSP windows yielded Bayes factors in favour of the null hypothesis (BF₀₁ = 1.6 and 2.1, respectively), indicating only weak evidence for the absence of congruency effects in these central components.

To examine expectancy conditions, a second set of models was fitted to the LF and FF conditions only. In the N450 window, there was a significant main effect of Condition (F(1, 1274) = 13.53, *p* = 0.0002), indicating overall amplitude differences between LF and FF, but neither Congruency (F(1, 1274) = 0.001, *p* = 0.98) nor the Condition × Congruency interaction (F(1, 1274) = 0.23, *p* = 0.63) were significant. The same pattern was found for the CSP window, with a main effect of Condition (F(1, 1274) = 5.342, *p* = 0.021) and no significant effects of Congruency (F(1, 1274) = 0.023, *p* = 0.878) or the Condition × Congruency interaction (F(1, 1274) = 0.25, *p* = 0.874) (Fig. 4B,C, respectively).

#### Mass-univariate analysis using permutation based cluster correction

**Fig. 5:**
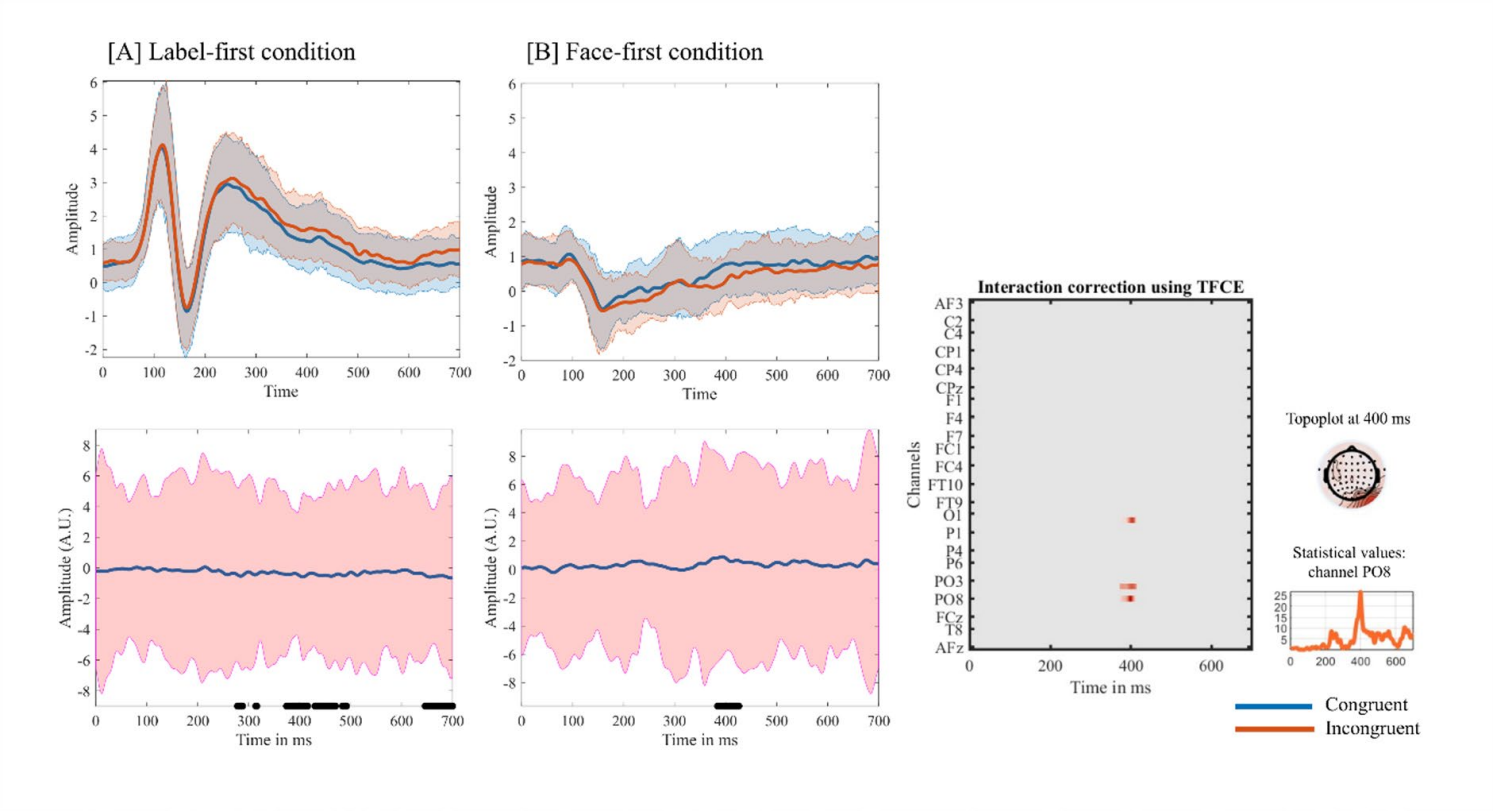
Condition × Congruency interaction over parieto-occipital sites using the Threshold Free Cluster Enhancement method. Grand average ERPs at PO8 for the Label-first (**A**) and Face-first (**B**) conditions are shown, showing congruent (blue) and incongruent (orange) trials with 95% confidence intervals. Corresponding difference waves (incongruent minus congruent) with 95% confidence intervals are shown below each plot; black horizontal segments indicate the time range of the significant LIMO cluster at PO8. The central panel displays the cluster-based permutation result for the Condition × Congruency interaction (TFCE-corrected, α = 0.05) across channels and time. The inset topography shows the scalp distribution of the interaction at 400 ms, and the lower right panel depicts the F-value time course at PO8.

The mass-univariate analysis in LIMO revealed a single significant Condition × Congruency interaction cluster over right parieto-occipital electrodes. After correction, the interaction reached its maximum at electrode PO8 around 400 ms post-stimulus, with the first significant frame at 374 ms, the last at 414 ms, and a peak F = 26.69 at 400 ms. The same cluster extended to neighbouring electrodes PO4 (max F = 26.50) and O2 (max F = 21.73) (Fig. 5).

### Primed expectancy effect: parietal attentional engagement related to conflict processing

Further analyses assessed how primed expectancy was processed in parietal sites and how it influenced conflict resolution, given the importance of the region in top-down control during anticipation^38^ and conflict resolution^13^.

#### CSP modulation by expectancy in parietal sites

Parietal ERPs were examined with linear mixed-effects models restricted to the expectancy conditions (LF and FF) in CSP mean amplitude from 600 ms to 800 ms. Models included Condition (LF, FF), Congruency, the Condition × Congruency interaction, and Run and Emotion as covariates. Neither the main effect of Congruency (F(1, 1274) = 0.002, *p* = 0.96) nor the Condition × Congruency interaction (F(1, 1274) = 2.36, *p* = 0.13) reached significance in CSP, and there was no reliable main effect of Condition (F(1, 1274) = 0.59, *p* = 0.44) on parietal amplitudes (Supplementary Analysis S4, Fig. S1). Bayesian paired-samples t-tests on CSP mean amplitudes comparing incongruent vs. congruent trials within each expectancy condition yielded Bayes factors in favour of the null hypothesis (LF: BF₀₁ = 2.6; FF: BF₀₁ = 2.9), indicating weak-to-moderate evidence for the absence of congruency effects in parietal CSP activity.

#### Parietal oscillatory activity during expectancy and conflict resolution

**Fig. 6:**
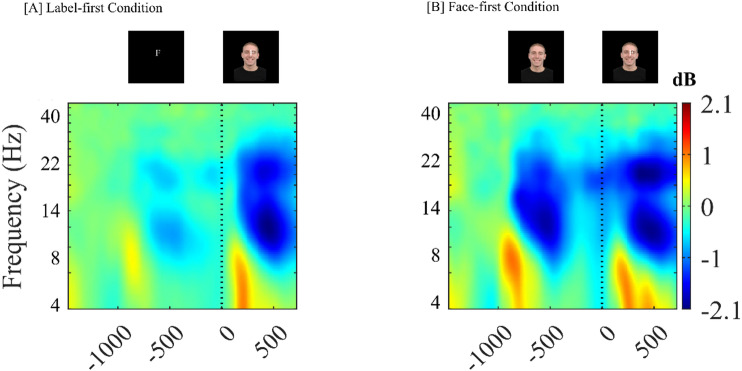
Time–frequency representations of event-related spectral perturbations (ERSPs) at the parietal cluster (P1, P2, Pz) for the Label-first (**A**) and Face-first (**B**) conditions. At − 1000 ms, the expectancy cue appears (letter in **A**, face in **B**); at 0 ms, the face and label overlap, marking conflict onset. The color scale (dB) indicates power changes relative to a common pre-event baseline. Consistent with the linear mixed-effects results, alpha and beta power show a Condition × Period interaction: in the Label-first condition, desynchronization is strongest during conflict, whereas in the Face-first condition, pronounced desynchronization is already present during the expectancy period with little additional change at conflict.

Parietal ERSPs were analyzed using a linear mixed-effects model with Condition (LF, FF), Period (expectancy vs. conflict), and Band (theta, alpha, beta) as fixed factors, and Congruency, Emotion, and Run as covariates. Across bands, there were robust main effects of Period (F(1, 7663) = 14.57, *p* = 0.0001) and Band (F(2, 7663) = 222.16, *p* = 1.63 × 10⁻⁹⁴), and a clear Condition × Period interaction (F(1, 7663) = 20.41, *p* = 6.3 × 10⁻⁶). Congruency and Emotion did not reach significance, and no Congruency interactions were observed (Fig. 6).

Follow-up models ran separately for each band showed partially distinct patterns. In the theta band, there were main effects of Condition (F(1, 2553) = 5.84, *p* = 0.016) and Period (F(1, 2553) = 7.00, *p* = 0.008), but the Condition × Period interaction was not significant (F(1, 2553) = 0.17, *p* = 0.68). Theta power was overall more suppressed in LF than FF (LF: −1.47 dB; FF: −1.05 dB), and more negative during expectancy than conflict (expectancy: −1.49 dB; conflict: −1.03 dB).

In the alpha band, there was no main effect of Condition (F(1, 2553) = 0.009, *p* = 0.924), but both Period (F(1, 2553) = 14.15, *p* = 0.0002), and Condition × Period interaction (F(1, 2553) = 17.11, *p* = 4.0 × 10⁻⁵) were significant. Overall, alpha power was more negative during conflict than expectancy (conflict: −3.48 dB; expectancy: −2.81 dB). However, this conflict-related desynchronization differed markedly by condition: in LF, alpha became substantially more negative at conflict (LF: conflict − 3.843 vs. expectancy − 2.427 dB), whereas in FF alpha power was already strongly suppressed during expectancy and changed very little at conflict (FF: conflict − 3.118 vs. expectancy − 3.186 dB).

A similar but stronger pattern emerged in the beta band. There was no main effect of Condition (F(1, 2553) = 0.06, *p* = 0.802), but Period showed a large effect (F(1, 2553) = 48.48, *p =* 4.23 × 10⁻¹²), and the Condition × Period interaction was significant (F(1, 2553) = 23.26, *p* = 1.49 × 10⁻⁶). Beta power was more negative during conflict than expectancy overall (conflict: −3.89 dB; expectancy: −3.05 dB), with a pronounced conflict-related desynchronization in LF (conflict − 4.166 vs. expectancy − 2.739 dB) and only a modest difference in FF (conflict − 3.612 vs. expectancy − 3.353 dB).

### Exploratory correlations between parietal oscillations and behavioral performance

To examine whether expectancy-related oscillatory changes were linked to performance, exploratory Pearson correlations were computed between accuracy and parietal alpha/beta indices, separately for the LF and FF conditions. In the LF condition, accuracy was not reliably associated with the magnitude of parietal beta desynchronization from expectancy to conflict (power difference between conflict and expectancy periods) (*r* = 0.20, *p* = 0.39), nor with the corresponding alpha index (*r* = 0.20, *p* = 0.39). Similarly, in the FF condition, neither beta (*r* = −0.22, *p* = 0.35) nor alpha (*r* = −0.22, *p* = 0.35) desynchronization was significantly related to accuracy. Exploratory correlations using a combined alpha–beta index (mean of the two bands) yielded essentially identical results (*r* = 0.20 for LF, *r* = −0.22 for FF, both *p* > 0.30). Complementary Bayesian correlation analyses yielded Bayes factors of BF₀₁ = 4.0 for the label-first condition and BF₀₁ = 3.7 for the face-first condition.

### Alpha and low beta source localization

**Fig. 7:**
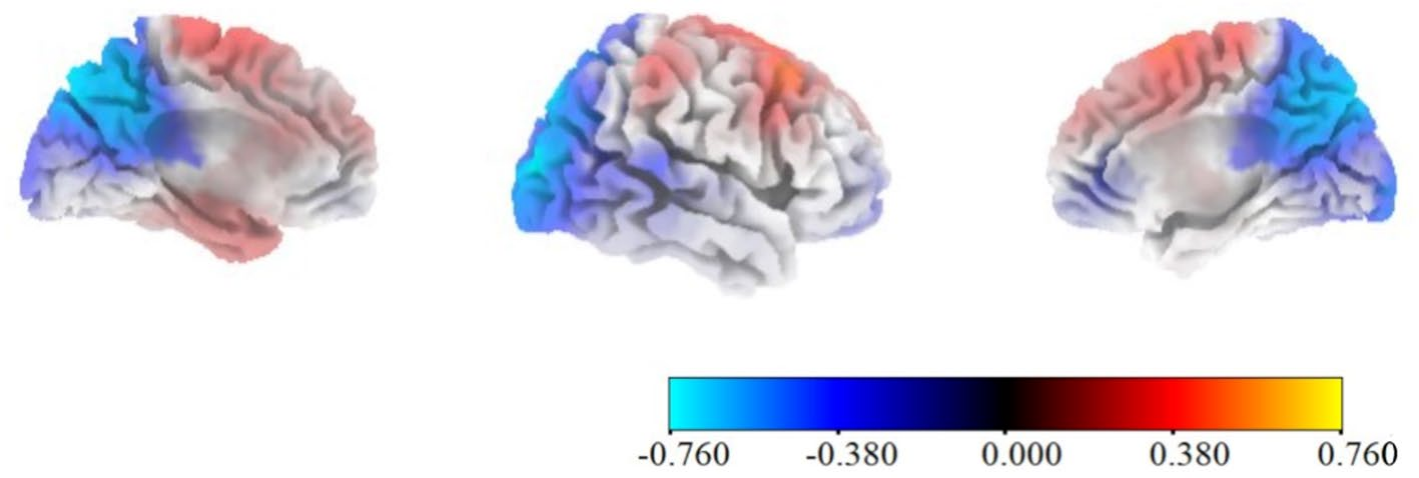
sLORETA source localization results. sLORETA signed *lnF* maps for the face-first (FF) vs. label-first (LF) expectancy contrast in low-beta (12–15 Hz) during 400–600 ms post-stimulus (conflict window). Inference used SnPM randomization (5000 permutations; paired) with FWER-corrected *p* = 0.05 critical lnF values: + 0.747 (FF > LF), − 0.742 (FF < LF); two-tailed = 0.783. The color scale encodes direction (warm = FF > LF; cool = LF > FF). A peak in BA7/precuneus at MNI (− 5, − 80, 50) reached *lnF* = − 0.760, significant one-tailed (*p* = 0.036, FWER-corrected) but not significant two-tailed (*p* = 0.076).

Focal significant differences in conflict processing (400–600 ms after stimulus onset) were found between the FF and LF conditions in the low-beta frequency range (12–15 Hz) in BA7/precuneus (MNI − 5, −80, 50). Using sLORETA’s signed *lnF* statistic, the peak was *lnF* = −0.760, indicative stronger decreases in low-beta power observed in the LF condition compared to the FF condition. This peak exceeded the FWER-corrected one-tailed threshold for the FF < LF direction (critical *lnF* = −0.742; *p* = 0.036, corrected) (Fig. 7). No significant differences in alpha and beta modulations were found between LF and FF in the expectancy period.

## Discussion

This study investigated whether the type of stimulus driven expectancy, using face versus non face cues, differentially influences conflict processing, in the context of emotion recognition. We hypothesized that expectancy would modulate conflict resolution, especially for face stimuli, owing to their unique processing priority in the brain^39–41^. Our findings provide evidence that primed expectancy differentially affects conflict-processing dynamics in a cue dependent manner, primarily at the level of oscillatory activity rather than classical central conflict ERPs.

We first aimed to replicate previously described conflict-related components, such as the N450^8,9^ and CSP^8^ using an Emotional Stroop task^42^, aligned with conflict monitoring theory^1^. In contrast to classic Stroop findings^41,43^, central N450 and CSP amplitudes did not show consistent congruency effects, either in the classical condition or when expectancy was introduced. Expectancy cue type did modulate central processing, but these shifts were not evident in conflict-related components. Instead, analysis threshold tree cluster enhancement and permutation correction showed a strong interaction between expectancy and congruence over at occipitoparietal sites, and expectancy-related differences emerged in parietal activity mostly in the oscillatory domain, which is more commonly linked to top-down attentional mechanisms and active conscious processing^12,44^.

Band specific time-frequency analysis helped clarify these effects by showing that parietal alpha and beta powers were strongly modulated by expectancy type and period. In the face-first condition, alpha and beta power were already strongly reduced during the expectancy generation period and changed very little during conflict, suggesting the posterior attentional resources were engaged early, prior to conflict itself. In contrast, in the label-first condition, desynchronization during expectancy was modest, whereas a pronounced decrease in alpha and beta power was observed during the conflict period, indicating a conflict-locked recruitment of attentional resources, possibly related to the less salient nature of the cue type (label-based expectancy compared to face expectancy). The altered temporal demands on attentional allocation, especially in the FF condition, are consistent with the idea that primes involving more salient stimuli, such as face cues, may reduce additional attentional and cognitive load during conflict processing^41,45^. This result is further supported by behavioral data, which showed a small but systematic increase in accuracy from the classical to the label-first and face-first conditions, suggesting a subtle improvement in performance when expectancy is manipulated.

The present oscillatory findings are consistent with Leocani et al. (2001), whose results suggest that beta power modulations are related to early attentional visual integration and consequently facilitate subsequent processing during the conflict period^46^. The stronger beta desynchronization during conflict in the LF condition contrasted with the relatively flat pattern shown in the FF condition, aligns with prior evidence that alpha and beta power reductions are more pronounced in situations demanding greater attentional recruitment, such as facial processing, particularly during the segregation of visual elements^47^. Source-level analysis further indicated that low-beta differences between the expectancy conditions during conflict were in the precuneus (Broadmann area 7), which has been linked to high-level processing tasks including attentional deployment^48^. This finding also aligned with the LIMO Expectancy x Congruency interaction results at the right parieto-occipital electrodes around 400 ms, supporting the view that expectancy type modulates conflict processing in posterior, high-level visual-attentional regions.

Regarding study limitations, although the use of a controlled laboratory task allowed for the precise investigation of specific cognitive mechanisms, it may limit the ecological validity of the findings, as real-world situations often involve more complex and dynamic environments. Future studies using multimodal imaging techniques, such as EEG combined with fMRI, could provide more detailed spatial and temporal insights into these processes. Furthermore, reaction times were not included as a behavioral measure, as participants were instructed to respond after stimulus offset and the appearance of a response cue. This procedure, despite being adopted to minimize motor-related EEG artifacts, excluded reaction time as a conflict processing index, since it would reflect movement execution speed rather than cognitive interference. In addition, exploratory correlations between parietal alpha and beta power and accuracy did not yield significant effects, a null result that should be interpreted cautiously given the relatively small sample size (*N* = 20). Finally, the sLORETA source localization method used in this study has known limitations in spatial resolution. Higher-resolution neuroimaging methods could help validate and extend our source-level findings.

Future work should examine expectancy-related conflict processing in clinical populations, such as individuals with anxiety or depression, who often exhibit expectancy biases^49^, and conflict processing alterations^50,51^. Incorporating more ecologically valid paradigms that better approximate real-life cognitive demands could also enhance the generalizability of the results.

## Conclusion

In summary, the present findings indicate that expectancy type, particularly face-driven expectancy, modulates conflict processing in Emotional Stroop contexts. Behaviorally, accuracy under expectancy conditions showed a modest but systematic tendency to increase, with a higher performance observed when expectations were based on facial cues. This pattern suggests that face-driven expectancy enhances conflict resolution by leveraging the brain’s prioritization of facial information, providing a slight advantage for performance.

At the neurophysiological level, classical central ERP components (N450 and CSP) did not show robust congruency effects in this paradigm, whereas clear differences emerged in the parietal oscillatory activity. Parietal alpha and beta power showed strong Condition x Period interactions, with label-based expectancy associated with relatively late, conflict-locked desynchronization and face-based expectancy associated with earlier desynchronization during the expectancy period. Source localization and LIMO analysis pointed to right parieto-occipital and precuneus regions as sites where expectancy type modulated post stimulus processing. Taken together, these results suggest that expectancy in Emotional Stroop contexts influences conflict processing by reshaping the time and focus of parietal attentional engagement, rather than by amplifying common conflict-related ERPs.

Our findings highlight the importance of expectancy in shaping cognitive processing, showing that face-driven expectations show a distinct deployment of attentional mechanisms and possibly enhancing performance during emotional recognition. Further research should explore this phenomenon in real-life settings and in clinical populations with altered conflict processing, offering further insights into socioemotional conflict resolution and intervention strategies.

## Supplementary Information

Below is the link to the electronic supplementary material.

Supplementary Material 1

## Data Availability

Data or materials for the experiments reported here are available upon reasonable request to the corresponding author, Miguel Castelo-Branco.

## Code availability

Research metadata and code supporting this publication are available at https://github.com/Daniel-Agostinho/ESP.

## Declarations

### Open practices statement

Data or materials for the experiments reported here are available upon reasonable request. None of the experiments were preregistered.
