# Supplementary material for: Face expectancy cues differentially modulate conflict processing driven by emotional incongruence: an EEG study
Source: Sci Rep. 2026 Jan 10;16:4457. doi: 10.1038/s41598-025-34447-9 (PMC12864925; doi:10.1038/s41598-025-34447-9)
Supplement: Supplementary file 1 — Supplementary Material 1 [file 41598_2025_34447_MOESM1_ESM.docx]

**Supplementary Material for:**

“Face expectancy cues differentially modulate conflict processing driven by emotional incongruence: An EEG study”

**Supplementary Analysis S1. Omission Rates Across Runs**

Table S1 describes the percentage of omission trials for each participant across the eight runs. Overall omission rates were low, with most participants showing omission percentages below 2% per run. A small subset of participants (P12, P14, P16) exhibited higher omission rates in some runs, typically between 3-6%. However, omission rates did not show a systematic trend across the experimental session, indicating stable task engagement.

**Table S1**

Percentage of omitted responses across runs for each participant

| Sum of Omissions | Column Labels |  |  |  |  |  |  |  |  |
| --- | --- | --- | --- | --- | --- | --- | --- | --- | --- |
| Row Labels | 1 | 2 | 3 | 4 | 5 | 6 | 7 | 8 | Grand Total |
| P01 | 3.09% | 1.55% | 0.52% | 1.03% | 0.00% | 0.00% | 2.06% | 0.52% | 8.76% |
| P02 | 0.52% | 0.00% | 0.52% | 0.00% | 0.52% | 0.52% | 0.52% | 0.00% | 2.58% |
| P03 | 0.00% | 0.00% | 0.00% | 0.00% | 0.00% | 0.00% | 0.00% | 0.00% | 0.00% |
| P04 | 0.00% | 0.00% | 0.00% | 0.00% | 0.00% | 0.00% | 0.00% | 0.00% | 0.00% |
| P05 | 0.00% | 0.00% | 0.00% | 0.00% | 0.00% | 0.52% | 0.00% | 0.00% | 0.52% |
| P06 | 0.00% | 0.00% | 0.00% | 0.00% | 0.00% | 0.00% | 0.00% | 0.52% | 0.52% |
| P07 | 1.55% | 2.06% | 0.00% | 3.09% | 1.03% | 2.58% | 2.06% | 4.12% | 16.49% |
| P08 | 1.55% | 0.00% | 0.52% | 0.00% | 0.00% | 0.00% | 1.03% | 0.00% | 3.09% |
| P09 | 5.67% | 2.06% | 2.06% | 2.06% | 1.55% | 1.55% | 2.58% | 3.61% | 21.13% |
| P10 | 1.55% | 0.00% | 0.52% | 0.52% | 1.03% | 0.00% | 0.00% | 1.55% | 5.15% |
| P11 | 1.55% | 0.00% | 2.58% | 4.12% | 2.06% | 2.58% | 2.06% | 1.55% | 16.49% |
| P12 | 1.03% | 0.52% | 0.52% | 0.52% | 0.00% | 2.06% | 0.00% | 0.00% | 4.64% |
| P13 | 0.52% | 0.00% | 0.00% | 0.00% | 0.00% | 0.52% | 0.00% | 0.00% | 1.03% |
| P14 | 0.52% | 0.00% | 0.00% | 0.00% | 0.00% | 0.00% | 0.00% | 0.00% | 0.52% |
| P15 | 0.00% | 0.00% | 0.00% | 0.00% | 0.00% | 0.00% | 0.00% | 0.52% | 0.52% |
| P16 | 0.00% | 0.00% | 0.52% | 0.00% | 0.00% | 1.03% | 0.52% | 1.55% | 3.61% |
| P17 | 0.52% | 0.52% | 0.00% | 1.03% | 0.00% | 0.00% | 0.00% | 0.00% | 2.06% |
| P18 | 0.00% | 0.00% | 0.00% | 0.52% | 0.52% | 1.03% | 0.00% | 0.52% | 2.58% |
| P19 | 1.03% | 1.55% | 0.52% | 0.52% | 1.55% | 1.55% | 1.03% | 1.03% | 8.76% |
| P20 | 0.00% | 0.00% | 0.52% | 0.52% | 0.52% | 0.00% | 0.00% | 0.00% | 1.55% |
| Total | 19.07% | 8.25% | 8.76% | 13.92% | 8.76% | 13.92% | 11.86% | 15.46% | 100.00% |

**Supplementary Analysis S2. Descriptive Reaction Time Statistics**

**Table S2**

Mean and Standard Deviation (SD) of Reaction Time (RT) and Accuracy by Condition

| Condition | Mean RT (s) | SD RT (s) | Accuracy (%) | Valid Trials | Omission Rate (%) |
| --- | --- | --- | --- | --- | --- |
| CL Congruent | 0.400 | 0.240 | 95.28 | 128.55 | 0.10 |
| CL Incongruent | 0.414 | 0.238 | 95.94 | 128.35 | 0.00 |
| LF Congruent | 0.290 | 0.204 | 96.02 | 128.40 | 0.05 |
| LF Incongruent | 0.293 | 0.211 | 96.72 | 128.45 | 0.00 |
| FF Congruent | 0.299 | 0.186 | 97.15 | 128.15 | 0.00 |
| FF Incongruent | 0.296 | 0.191 | 97.46 | 128.40 | 0.00 |

**Supplementary Analysis S3. Accuracy between conditions**

Descriptive statistics indicated high task performance across all conditions. The percentage of valid trials was consistently above 95%, suggesting participant engagement and accurate responding.

**Table S3**

Minimum, Maximum, Mean and Standard Deviation (SD) of Accuracy (%) by Condition

| **Condition** | **Hit rate (%)** | | | |
| --- | --- | --- | --- | --- |
|  | **Minimum** | **Maximum** | **Mean** | **SD** |
| **CL: Congruent trials** | 84 | 100 | 95.3 | 3.83 |
| **CL: Incongruent trials** | 84 | 100 | 95.9 | 4.17 |
| **LF: Congruent trials** | 87 | 100 | 96.0 | 3.86 |
| **LF: Incongruent trials** | 89 | 100 | 96.7 | 3.55 |
| **FF: Congruent trials** | 85 | 100 | 97.2 | 3.38 |
| **FF: Incongruent trials** | 90 | 100 | 97.4 | 3.12 |

**Supplementary Analysis S4. Parietal CSP in the expectancy conditions**


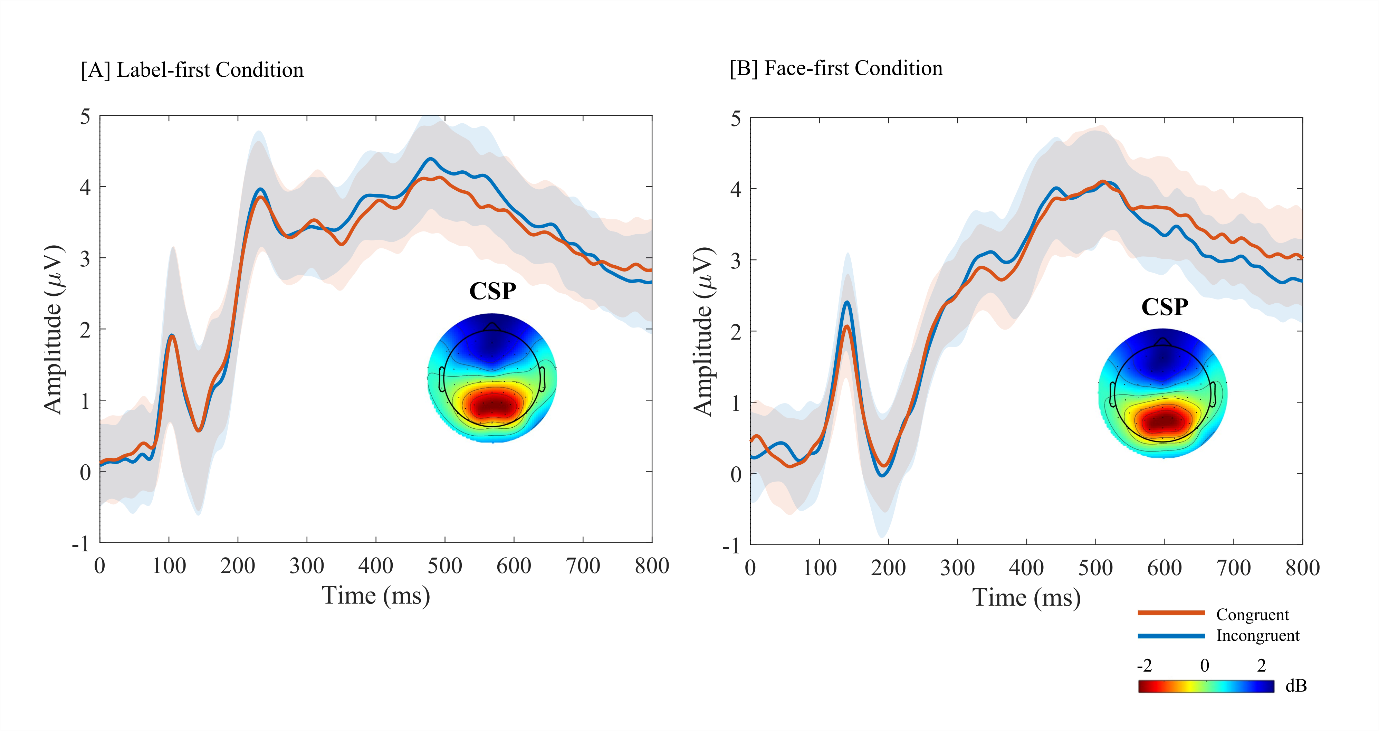


**Figure S1.** Parietal CSP in the conditions where expectancy was manipulated. Grand-average ERPs at the parietal cluster (P1, P2, Pz) for the Label-first (LF), and Face-first (FF) conditions. Time 0 ms marks the conflict onset, corresponding to the face–label pair. Blue and orange traces show congruent and incongruent trials, respectively; the shaded regions represent the standard error (SE) of the mean across participants. Scalp topographies for the CSP (600 ms to 800 ms) windows are shown. Mean amplitudes in these predefined windows were used in the linear mixed-effects models reported in the Results section.
